# A World Full of Stereotypes? Further Investigation on Origin and Gender Bias in Multi-Lingual Word Embeddings

**DOI:** 10.3389/fdata.2021.625290

**Published:** 2021-06-03

**Authors:** Mascha Kurpicz-Briki, Tomaso Leoni

**Affiliations:** Department of Engineering and Information Technology, Institute for Data Applications and Security IDAS, Bern University of Applied Sciences, Biel/Bienne, Switzerland

**Keywords:** word embeddings, fairness, digital ethics, natural language processing, training data, language models, gender, bias

## Abstract

Publicly available off-the-shelf word embeddings that are often used in productive applications for natural language processing have been proven to be biased. We have previously shown that this bias can come in different forms, depending on the language and the cultural context. In this work, we extend our previous work and further investigate how bias varies in different languages. We examine Italian and Swedish word embeddings for gender and origin bias, and demonstrate how an origin bias concerning local migration groups in Switzerland is included in German word embeddings. We propose BiasWords, a method to automatically detect new forms of bias. Finally, we discuss how cultural and language aspects are relevant to the impact of bias on the application and to potential mitigation measures.

## 1. Introduction

Algorithms and data-based applications are highly sensitive to bias in the underlying training data and can therefore contain risks of discrimination for different groups of the society. Different types of application learning from existing data are affected by this problem: Problems of racism have been detected in image classification applications. For example, Google's mobile photo application labeled African-Americans as *gorillas* or an automated passport application asked applicants of Asian descent to open their eyes (Howard and Borenstein, 2018). Furthermore, gender discrimination in a recruiting program of a large tech company (Dastin, 2018) and fairness problems in a risk assessment software for criminal offenders (Angwin et al., 2016) have been identified, just to mention a few cases.

In particular, in the area of Natural Language Processing (NLP) such kind of problems have been identified. For example, it has been show that automatic translation utilities rely on gender stereotypes when translating professions (Kurpicz-Briki, 2020) and that Wikipedia has a gender bias concerning how often and in which way men and women are described (Wagner et al., 2015, 2016). Learning from existing real-world data and making smart decisions based on such data amplifies cultural stereotypes (Barocas and Selbst, 2016) due to historical bias encoded inside the data. It is, therefore, relevant to provide metrics to measure and mitigate the bias in training data (Sun et al., 2019).

Examples of such a bias can easily be verified by tools in our daily life such as automatic translation utilities. By translating the English sentence *I am looking for an Engineer. Can you help?* to German, the male form of the word engineer (*Ingenieur*) is used. On the other side, when translating the English sentence *I am looking for a nurse. Can you help?* to German, the female word for nurse is used (*Krankenschwester*). In German, the words *nurse* or *engineer* could also be translated to the female or male form, respectively, being *Ingenieurin* or *Krankenpfleger*. However, based on stereotypes of the society deeply integrated inside our language and language models, this option is not proposed and it is assumed that the nurse is a woman, and the engineer is a man.

In particular, pre-trained language models, such as word embeddings have been shown to be biased in different work (e.g., Bolukbasi et al., 2016; Caliskan et al., 2017; Kurpicz-Briki, 2020). Even in more recent contextualized language models such as Google's BERT model (Devlin et al., 2018), indications of bias have been detected (Kurita et al., 2019).

Word embeddings are vector representations of words, trained on co-occurrences of words in large text corpora. Words with vectors that are closer together, i.e., having a smaller vector distance, typically have a similar meaning and can, therefore, be used to represent relationships between words. When a domain-specific word model is required, such vectors are trained on specific corpora. However, in many applications the publicly available word embeddings are sufficient. Common publicly available word embeddings are word2vec (Mikolov et al., 2013a,b,c), fastText (Bojanowski et al., 2017; Grave et al., 2018), and GloVe (Pennington et al., 2014).

Since such pre-trained language models are easily and publicly available on the internet (Hapke et al., 2019), they are nowadays often already used in production of natural language processing applications. The bias implicitly encoded inside those language models can, therefore, be assumed to be in production as well.

To measure bias in word embeddings, the Word Embedding Association Test (WEAT) has been proposed (Caliskan et al., 2017) and applied and/or adapted in different other works (e.g., Karve et al., 2019; May et al., 2019; Zhou et al., 2019). The WEAT is based on a method borrowed from the field of Psychology, the so-called Implicit Association Test (IAT) (Greenwald et al., 1998), which is used to measure implicit bias in humans. Along with the presentation of the WEAT method, the authors have extensively shown the existence of implicit stereotypes in GloVe and word2vec word embeddings for the English language (Caliskan et al., 2017). The WEAT is based on two groups of attribute words and two groups of target words. By using the cosine similarity between the word embeddings of these word groups (as replacement for the reaction time used in the IAT), a statistical test is applied: The null hypothesis is that there is no difference between the two sets of target words with regard to relative similarity to the two sets of attribute words.

In particular, the authors of the WEAT confirmed that the same stereotypes that were detected in humans subjects using the IAT in Psychology are encoded also in word embeddings. This concerned stereotypes regarding both gender and origin. For example, their experiments have shown that there is a bias concerning family words (e.g., *home, family, relatives*,.) and career words (e.g., *executive, management, professional*,.) toward male and female first names. In a similar way, they showed that there is bias when using pleasant and unpleasant words in combination with European-American and African-American first names.

Since the WEAT and numerous adaptations and further applications are based on English word embeddings, not much information on bias for word embeddings in other languages is available yet. However, word embeddings, and in particular fastText word embeddings (Bojanowski et al., 2017) are available in 157 languages (Grave et al., 2018). It has also been shown that working with other languages than English, the WEAT is challenged by language-specific particularities such as grammatical gender (Zhou et al., 2019). Another work (McCurdy and Serbetci, 2017) (based on training data from OpenSubtitles corpus, Lison and Tiedemann, 2016) has explored whether word embeddings in languages with grammatical gender show the same topical semantic bias as in English, and reliable effects were found for Dutch. For Italian, in particular, gender stereotypes in job-related word embeddings have been found (Biasion et al., 2020), using the WEAT method for validation.

Our recent work (Kurpicz-Briki, 2020) has applied WEAT to German and French word embeddings and proven that there is discrimination against origin and gender in both languages. In particular, we have shown that real-world bias is reflected, and society stereotypes from the Eighteenth century can still be found in current word embeddings. Our work has also found that cultural stereotypes can be different among different languages and, therefore, each language needs to be examined carefully. Other research has confirmed that such stereotypes are reflected in the structure of natural language semantics, and therefore, linguistic associations shape people's implicit judgments (Lewis and Lupyan, 2020).

In this work, we further investigate on how bias and stereotypes of our society are encoded in current word embeddings in different languages. Therefore, we extend our previous work on German and French to additional languages. We considered the same experiments on word embeddings in Swedish and Italian. Based on our previous findings, that bias in word embeddings comes in different forms depending on the language, we also developed two new experiments based on word sets reflecting the situation of specific minorities in Switzerland.

Currently, such culturally specific biased word sets had to be identified based on sources such as real-world data or statistics of the population. In this paper, we propose BiasWords, an automatic approach to detect such biased word sets by exploring the vector space.

Based on these experiments, we want to address the following research questions:

- Can known gender and origin bias found in pre-trained word embeddings be confirmed for Italian and Swedish?
- Is an origin bias concerning specific minorities in Switzerland reflected in German word embeddings?
- Can we detect automatically new biased word sets by exploring the vector space?

We, first, present the used datasets and provide a detailed experiment description on how the WEAT experiments were adapted for Italian and Swedish. We, then, present the new word sets for the detection of origin bias in Switzerland which were identified in this work. We then present BiasWords, a new method to automatically detect new forms of bias in word embeddings. After presenting the results of our experiments, we discuss potential reasons with regard to related work and the impact of such cultural and linguistic differences on the future work in the field.

## 2. Materials and Methods

### 2.1. The WEAT Method

In the work presented in this paper, we apply the WEAT (Caliskan et al., 2017) to multi-lingual word embeddings in different languages.

The WEAT method (Caliskan et al., 2017), including its terminology, is based on the IAT (Greenwald et al., 1998) from psychology. The IAT measures a person's subconscious association between concepts and therefore gives a measure for implicit bias. It is a computer-based measure that asks users to rapidly categorize two target concepts with an attribute. These questions are based on combining possible answers to parallel non-biased questions, and therefore implicit stereotypes can be measured. Easier pairing (i.e., shorter reaction time) is interpreted as as stronger association between the concepts.

Such IAT experiments consist of two sets of *target words*, for example (*math, algebra*, ...) and (*art, poetry*, ...). Furthermore, two sets of *attribute words* are defined, for example (*man, male*, ...) and (*woman, female*, ...)

In WEAT, the distance between vectors corresponds to the reaction time in IAT. As a measure of distance between the vectors, the cosine similarity between the vectors is computed.

The null hypothesis is that there is no difference between the two sets of target words with regard to relative similarity to the two sets of attribute words (i.e., there is no bias between the genders regarding the target word groups).

The WEAT test can be formalized as follows (Caliskan et al., 2017): *X* and *Y* are the two sets of target words of equal size. *A* and *B* are the two sets of attribute words. *s*(*X, Y, A, B*) is the test statistics.

(1) $$\begin{array}{r} s\left(X,Y,A,B\right)=\underset{x\text{∈}X}{\sum }s\left(x,A,B\right)-\underset{y\text{∈}Y}{\sum }s\left(y,A,B\right) \end{array}$$

where

$$\begin{array}{l} s\left(w,A,B\right)=\\ mean_{a\text{∈}A}cos\left(\vec{w},\vec{a}\right)-mean_{b\text{∈}B}cos\left(\vec{w},\vec{b}\right) \end{array}$$

*s*(*w, A, B*) measures the association of *w* with the attribute. *s*(*X, Y, A, B*) measures the differential association of the two sets of target words with the attribute. In the equation, $cos\left(\vec{a},\vec{b}\right)$ defines the cosine of the angle between the vectors $\vec{a}$ and $\vec{b}$, which we use to measure the distance between the two vectors. In WEAT, a permutation test is used to measure the (un)likelihood of the null hypothesis, i.e., they compute the probability that a random permutation of the attribute words would produce the observed (or greater) difference in sample means.

{(*X*_*i*_, *Y*_*i*_)} denotes all the partitions of *X*∪*Y* into two sets of equal size. The one-sided *p*-value is then defined as Caliskan et al. (2017):

(2) $$\begin{array}{r} Pr_{i}\left[s\left(X_{i},Y_{i},A,B\right)>s\left(X,Y,A,B\right)\right] \end{array}$$

In our implementation, instead of the full permutation test, we implemented a randomization test with 100'000 iterations, following (Chaloner and Maldonado, 2019). The effect size is computed as Cohen's *d* (as for the original IAT). The effect size *d* is computed as Caliskan et al. (2017).

(3) $$\begin{array}{r} \frac{mean_{x\text{∈}X}s\left(x,A,B\right)-mean_{y\text{∈}Y}s\left(y,A,B\right)}{stddev_{w\text{∈}X\cup Y}s\left(w,A,B\right)} \end{array}$$

Our implementation of WEAT has been previously verified by reproducing the results for English word embeddings of the original WEAT paper (Kurpicz-Briki, 2020). In the experiments described in this paper, we extend our previous work for German and French to additional languages. We adapted and/or translated the selected WEAT experiments to Italian and Swedish to investigate whether an origin and/or gender bias can be identified.

### 2.2. Datasets

In the experiments described in this paper, we used fastText word embeddings (Bojanowski et al., 2017). Pre-trained models of fastText word embeddings are available in multiple languages (Grave et al., 2018), including Italian and Swedish. Word embeddings trained with fastText provide an advantage to word2vec. Instead of predicting the surrounding words, the surrounding n-character grams are predicted. This results in the advantage that rare words can be handled much better (Hapke et al., 2019).

In this work, we focused on pre-trained word embeddings as they are often used in practice NLP applications. Such word models are publicly available on the internet and can be used for different projects, unless a domain-specific word model is required (Hapke et al., 2019). Therefore, bias contained in such off-the-shelf word embeddings can easily propagate to numerous applications. Previous work in the field did also consider pre-trained word embeddings, e.g., using GloVe and word2vec (Caliskan et al., 2017) or fastText (Karve et al., 2019).

The word embeddings used in our experiment had 300 dimensions and were trained on CommonCrawl and Wikipedia. Table 1 compares the dataset sizes. The first row contains the sizes of each officially downloadable compressed archive containing all word vectors for each language^1^. The second row shows the number of all Wikipedia articles for each individual language, while the third row contains the share of the Common Crawl corpus for each language. All Wikimedia stats come from the official Wikimedia List of Wikipedias^2^. The Common Crawl share was obtained from the CommonCrawl statistics tool^3^. These values describe on how much data the word embeddings for each language have been trained on.

**Table 1:** Size comparison of the different fastText word embeddings used in this paper.

**Source**	**English**	**German**	**French**	**Italian**	**Swedish**	**Romansh**
Zip file (GB)	1.33	1.28	1.29	1.27	1.25	0.0654
Wikipedia articles	6′161′892	2′481′753	2′251′004	1′636′253	3′675′493	3′695
CC share percentage	43.7532	5.5476	4.6213	2.4019	0.7487	0.0014

In the new experiments investigating origin bias in Switzerland, the German word embeddings from fastText are used.

### 2.3. Bias Detection: Experimental Setup

#### 2.3.1. WEAT5 - Local/Foreign Names vs. (Un)pleasant Words

The WEAT5 experiment from the original paper considers European-American and African-American names and pleasant/unpleasant words. We translated (and where necessary adapted) the original experiment to Italian and Swedish. Table 2 shows the complete listing of the selected words. In the following paragraphs, we describe the particular selection and translation of words for each language:

**Table 2:** The terms from the original WEAT5 experiment (Caliskan et al., 2017) and our adaptations/translations to Italian and Swedish.

**Group**	**WEAT5-ori**	**WEAT5-ita**	**WEAT5-swe**
Group 1	Brad, Brendan, Geoffrey, Greg, Brett, Jay, Matthew, Neil, Todd, Allison, Anne, Carrie, Emily, Jill, Laurie, Kristen, Meredith, Sarah	Andrea, Francesco, Alessandro, Matteo, Luca, Martina, Alessia, Giulia, Chiara, Sara	Lucas, Liam, William, Elias, Noah, Hugo, Oliver, Oscar, Adam, Alice, Olivia, Astrid, Maja, Vera, Ebba, Ella, Wilma, Alma
Group 2	Darnell, Hakim, Jermaine, Kareem, Jamal, Leroy, Rasheed, Tremayne, Tyrone, Aisha, Ebony, Keisha, Kenya, Latonya, Lakisha, Latoya, Tamika, Tanisha	Kevin, Denise, Thomas, Aaron, Jennifer, Anita, Gabriell, Michael, Thiago, Ivan, Iris, Santiago, Igor, William, Sharon, Abigail	Muhammad, Sai, Advik, Rudra, Arya, Saanvi, Maryam, Amaira, Hussain, Omar, Usman, Khadija, Zuleikha, Fatima, Farid, Hassan, Amira, Iman
Pleasant	Joy, love, peace, wonderful, pleasure, friend, laughter, happy	Gioia, amore, pace, incredibile, piacere, amichevole, ridere, felice	glädje, förälskad, fred, ofattbar, nöje, vän, skratt, glad
Unpleasant	Agony, terrible, horrible, nasty, evil, war, awful, failure	Agonia, terribile, orribile, sgradevole, crudele, guerra, terrificante, fallimento	kval, hemsk, förskräcklig, otrevlig, ondska, krig, skrämmande, fel

**Italian** - To identify the most common Italian local names, data from the Italian National Statistics Institute (*Istituto Nazionale di Statistica*) was available^4^. Unfortunately, no direct data for names of foreign origin was available. We, therefore, used data from a website that proposes potential baby names by different categories and selected the most clicked names of foreign origin from the website's statistics^5^. The pleasant and unpleasant words were translated from English. We replaced the word *wonderful* with *incredibile* (Englisch: *incredible*) to better suit the Italian language, as proposed by the native speaker. Due to the same reason, we replaced *nasty* with *sgradevole* (English: *unpleasant*) and *awful* with *terrificante* (English: *terrifying*). However, translations always contain subjectivity. Therefore, we further validated the impact of our choice as follows: we repeated the experiment by replacing *incredibile* by other options: *magnifico, fantastico*, and *meraviglioso*, as well as the female forms *magnifica, fantastica*, and *meravigliosa*.

**Swedish** - For the typical Swedish names for men and women, we considered the data made available through Statistics Sweden^6^. Since no list of popular names of different origins in Sweden was available, we compiled a list of the most popular names of Afghan, Indian, and Syrian origin, proportional to the percentage of the population. The pleasant and unpleasant words were translated to Swedish from the original experiments in English.

#### 2.3.2. WEAT6 - Male/Female Names vs. Career/Family Words

The WEAT6 experiment from the original paper considers American male/female names and career/family words. We translated (and where necessary adapted) the original experiment to Italian and Swedish. Table 3 shows the complete listing of the selected words. In the following paragraphs, we describe particularities regarding the selection and translation of words for each language:

**Table 3:** The terms from the original WEAT6 experiment (Caliskan et al., 2017) and our adaptations/translations to Italian and Swedish.

**Group**	**WEAT6-ori**	**WEAT6-ita**	**WEAT6-ita-ch**	**WEAT6-swe**
Group 1	John, Paul, Mike, Kevin, Steve, Greg, Jeff, Bill	Andrea, Francesco, Alessandro, Matteo, Luca, Lorenzo, Marco, Davide, Simone, Giuseppe	Marco, Luca, Andrea, Giuseppe, Alessandro, Francesco, Antonio, Roberto	Lucas, Liam, William, Elias, Noah, Hugo, Oliver, Oscar
Group 2	Amy, Joan, Lisa, Sarah, Diana, Kate, Ann, Donna	Martina, Alessia, Giulia, Chiara, Sara, Francesca, Frederica, Giorgia, Anna, Elisa	Maria, Anna, Daniela, Sara, Laura, Elena, Francesca, Giulia	Alice, Olivia, Astrid, Maja, Vera, Ebba, Ella, Wilma
Career	Executive, management, professional, corporation, salary, office, business, career	dirigente, gestione, professionale, corporazione, salario, ufficio, affari, carriera	dirigente, gestione, professionale, corporazione, salario, ufficio, affari, carriera	ledare, företagsledning, professionell, bolag, lön, byrå, företag, karriär
Family	Home, parents, children, family, cousins, marriage, weddings, relatives	casa, genitori, bambini, famiglia, cugini, matrimonio, nozze, parenti	casa, genitori, bambini, famiglia, cugini, matrimonio, nozze, parent	hem, föräldrar, barn, familj, kusiner, gifte, bröllop, släkt

*For Italian, a Swiss and an Italian version of the experiment was used*.

**Italian** - For Italian, two different experiments were conducted. Once, we considered the most often used names in Italy from the Italian National Statistics Institute, as in the previous experiment. In the second experiment, we considered the most common names in the Italian-speaking region of Switzerland, provided by the Swiss Federal Statistics Office^7^. The career and family words were in both cases translated from English to Italian. As opposed to English, in Italian often male and female versions of professions exist (e.g., *il professore, la professoressa*). However, the female version is not used consequently, often the generic male version of the word is used for both genders. To avoid this having an impact on our results, we picked words in the word set that do not express gender (e.g., *il/la dirigente*). To further validate the impact of such personal words, we repeated the experiment by replacing the word *dirigente* with the word *successo* (engl: *success*).

**Swedish** - For the typical Swedish names for men and women, we considered the data made available through Statistics Sweden^8^. We translated the English words from the original experiments to Swedish. However, the word *corporation* was replaced with the Swedish translation of *company*, and *leader* was used instead of *executive* to better match the Swedish language.

#### 2.3.3. WEAT7 - Math/Arts vs. Male/Female Words

We translated (and where necessary adapted) the WEAT7 experiment from the original paper which considered math and arts words, and male and female words. Table 4 shows the complete listing of the selected words. In the following paragraphs, we describe particularities regarding the selection and translation of words for each language:

**Table 4:** The terms from the original WEAT7 experiment (Caliskan et al., 2017) and our adaptations/translations to Italian and Swedish.

**Group**	**WEAT7-ori**	**WEAT7-ita**	**WEAT7-swe**
Math	Math, algebra, geometry, calculus, equations, computation, numbers, addition	matematica, algebra, geometria, calcolo, equazioni, computo, numeri, addizione	matematik, algebra, geometri, kalkyl, ekvation, beräkning, siffror, addition
Arts	Poetry, art, dance, literature, novel, symphony, drama, sculpture	poesia, arte, danza, letteratura, romanzo, sinfonia, dramma, scultura	poesi, konst, dans, litteratur, roman, symfoni, drama, skulptur
Male terms	Male, man, boy, brother, he, him, his, son	maschio, uomo, ragazzo, fratello, egli, lui, suo, figlio	manlig, man, pojke, bror, han, honom, hans, son
Female terms	Female, woman, girl, sister, she, her, hers, daughter	femmina, donna, ragazza, sorella, ella, lei, suo, figlia	kvinnlig, kvinna, flicka, syster, hon, henne, hennes, dotter

**Italian** - The math/arts and male/female words were translated to Italian.

**Swedish** - The math/arts and male/female words were translated to Swedish.

#### 2.3.4. WEAT8 - Science/Arts vs. Male/Female Words

We translated (and where necessary adapted) the WEAT8 experiment from the original paper which is considering science and arts words, and male and female words. Table 5 shows the complete listing of the selected words. In the following paragraphs, we describe any particularities regarding the selection and translation of words for each language:

**Table 5:** The terms from the original WEAT8 experiment (Caliskan et al., 2017) and our adaptations/translations to Italian and Swedish.

**Group**	**WEAT8-ori**	**WEAT8-ita**	**WEAT8-swe**
Science	Science, technology, physics, chemistry, Einstein, NASA, experiment, astronomy	scienza, tecnologia, fisica, chimica, Einstein, NASA, esperimento, astronomia	vetenskap, teknologi, fysik, kemi, Einstein, NASA, försök, astronomi
Arts	Poetry, art, Shakespeare, dance, literature, novel, symphony, drama	poesia, arte, Shakespeare, dansa, letteratura, romanzo, sinfonia, dramma	poesi, konst, Shakespeare, dans, litteratur, roman, symfoni, drama
Male terms	Brother, father, uncle, grandfather, son, he, his, him	fratello, padre, zio, nonno, figlio, egli, suo, lui	bror, far, farbror, farfar, son, han, hans, honom
Female terms	Sister, mother, aunt, grandmother, daughter, she, hers, her	sorella, madre, zia, nonna, figlia, ella, suo, lei	syster, mor, moster, mormor, dotter, hon, hennes, henne

**Italian** - The math/arts and male/female words were translated to Italian. We considered NASA, Einstein, and Shakespeare as internationally known words and therefore kept these words for all the languages.

**Swedish** - The science/arts and male/female words were translated to Swedish. However, Swedish does not have generalized terms for most relatives, it is always declared what side they are from, thus *uncle* becomes *morbror* (mother's brother) or *farbror* (father's brother). We used the father's side for the male terms and the mother's side for the female terms in our experiments.

#### 2.3.5. Origin Bias in Switzerland

As shown in our previous work, the form of bias in word embeddings in different languages can vary (Kurpicz-Briki, 2020). We, therefore, designed additional experiments to identify origin bias in German word embeddings, based on word groups, in particular, representing the situation in Switzerland. Table 6 shows the exact wording of the experiments. The next paragraphs give detailed information about the experimental setup for each experiment.

**Table 6:** The terms from the new experiments investigating origin bias in Switzerland on German word embeddings.

**Group**	**WEAT5-origin**	**WEAT6-origin-m**	**WEAT6-origin-f**
Group 1	Peter, Daniel, Hans, Thomas, Andreas, Martin, Markus, Michael, Maria, Anna, Ursula, Ruth, Monika, Elisabeth, Verena, Sandra	Peter, Daniel, Hans, Thomas, Andreas, Martin, Markus, Michael	Maria, Anna, Ursula, Ruth, Monika, Elisabeth, Verena, Sandra
Group 2	Fatime, Bajram, Emine, Bekim, Aferdita, Valon, Egzon, Luljeta, Stojan, Marija, Snežana, Aleksandar, Mehmet, Mustafa, Fatma, Ayşe	Valon, Egzon, Stojan, Aleksandar, Mehmet, Mustafa, Bajram, Bekim	Fatime, Emine, Aferdita, Luljeta, Marija, Snežana, Fatma, Ayşe
Positive	Spass, Liebe, Frieden, wunderbar, Freude, Lachen, glücklich	Führungskraft, Verwaltung, beruflich, Konzern, Gehalt, Büro, Geschäft, Werdegang	Führungskraft, Verwaltung, beruflich, Konzern, Gehalt, Büro, Geschät, Werdegang
Negative	Qual, furchtbar, schrecklich, übel, böse, Krieg, scheusslich, Versagen	versagen, Abbruch, Armut, arbeitslos, Sozialhilfe, untätig, unqualifiziert, Last	versagen, Abbruch, Armut, arbeitslos, Sozialhilfe, untätig, unqualifiziert, Last

**WEAT5-origin** - We adapted the WEAT5 experiment to cover the different groups of population in Switzerland. We have previously shown that there is bias when considering Swiss names and names of foreign origin in general (Kurpicz-Briki, 2020), and therefore we this time considered three concrete groups of names with foreign origin. According to the Swiss Federal Statistical Office^9^, the three most common Non-EU countries of origin (that are still in the European continent, which excludes origins such as Africa, the Americas, etc.) of Switzerland's inhabitants are Kosovo, North Macedonia, and Turkey. We, therefore, used common names in proportional combination of these three countries (at 8:4:4), using local sources^10^,^11^,^12^. We selected originally Swiss German names by using the eight most common names of the German part of Switzerland for women and men based on data from the Swiss Federal Statistical Office^13^.

**WEAT6-origin** - Based on the WEAT6 experiment from the original paper, we designed a new experiment to investigate a potential origin bias regarding business for both men and women. We, therefore, considered the Swiss names and names of foreign origin as described in the previous paragraph, conducting the experiment for women *WEAT6-origin-f* and men *WEAT6-origin-m* separately. We considered positive and negative words regarding business and inclusion in the system. As positive words, the business terms from the original WEAT6 experiment were used. As negative words, a list of words including *Sozialhilfe, Abbruch*, or *unqualifiziert* was compiled (English: *welfare, dropping out, and unqualified*). This experiment aims to investigate whether there is a bias toward people of different origin with regard to inclusion in society and labor market. All the selected words are gender-neutral, so that the same list of words was used in both experiments.

### 2.4. BiasWords - Automatic Bias Detection

The word sets used to detect bias in word embeddings are based on the original experiments from the IAT from psychology (Caliskan et al., 2017). We have previously shown that additional word sets can be identified by translating the word sets to other languages (which does not always work) or using real-world data, such as study choices or stereotypes from the eighteenth century (Kurpicz-Briki, 2020). However, the identification of these word sets has been a manual process. In this paper, we present BiasWords, a method to automatically detect new potentially biased word sets by exploring the vector space.

As a proof of concept, we are using German word embeddings to demonstrate our approach. However, the same procedure could be applied to word embeddings of any language. We select the well-known word sets from literature (that show bias) and explore the vector space around each word in these word sets. Given the fact that word vectors being closer together have similar meaning, we expect that words being close to biased words might themselves also be biased.

In this proof of concept, we considered the experiments from our previous work, i.e., WEAT 5 to 8 and GER-1/GER-2 (Kurpicz-Briki, 2020). For each word in each of the word sets, the 20 nearest neighbors are identified and a selection procedure is applied: the part-of-speech tag must be compatible, and the words must differ by at least three letters. The part-of-speech tag is considered as compatible, when it is either the same, or an adjective (*ADJ*) with an adverb (*ADV*), or a noun (*NOUN*) with a proper name (*PROPN*). We require the difference of at least three letters to avoid other forms of the same word to be considered, e.g., the same word containing a typo. We did a data cleanup, removing empty string, duplicates, and replacing the letter ß by double s, as it is not common in all German speaking regions.

In particular, for the generated categories containing male (e.g., *männlich, Bruder*, and *Sohn*- engl.: *male, brother*, and *son*) or female (e.g., *weiblich, Schwester*, and *Tochter*- engl.: *female, sister*, and *daughter*) words, some words from the opposite sex where included and had to be removed. Due to the fact that family matters are close in vector space, there is an overlap and words such as *Ehemann* (engl.: *husband*) is close to both male and female words. Neutral words that appeared in such categories (e.g., *gutaussehend*- engl.: *handsome*) were kept. Words containing spelling mistakes were also kept.

After this step, for each category of each of the existing word sets, there is a list of other words that are candidates for the new word set. We, then, chose random words from each list, the same number as the size of the original word sets. We validated the newly created word sets using the WEAT method previously explained.

## 3. Results

In this section, the results of the previously described experiments are reported. In this work, we consider a statistically significant bias if the *p*-value is below 0.05, following the other work in the field (Caliskan et al., 2017; Chaloner and Maldonado, 2019).

### 3.1. Multi-Lingual Gender and Origin Bias

We confirmed a bias for the WEAT5 experiment for Italian and Swedish word embeddings. In the original WEAT5 experiment (Caliskan et al., 2017), African-American and European-American names were considered. We adapted the experiment with typical foreign names in the considered countries and could confirm the bias with regard to these names. In our previous work (Kurpicz-Briki, 2020), this had already been confirmed for German and French word embeddings.

For Italian, we validated our choice of the word *incredibile*, as mentioned previously, by replacing it with other options in male and female form. We could confirm the bias also by using *meraviglioso* (*p*-value: 0.00995, effect size: 0.586), *meravigliosa* (*p*-value: 0.00097, effect size: 0.819), *magnifico* (*p*-value: 0.00934, effect size: 0.598), *magnifica* (*p*-value: 0.00254, effect size: 0.674), *fantastico* (*p*-value: 0.01256, effect size: 1.183), and *fantastica* (*p*-value: 0.00497, effect size: 0.308).

The WEAT6 experiment evaluates bias between male and female names, and career and family words. Our experiments identified a bias in Italian word embeddings, for both the names from Italian-speaking Switzerland and from Italy. We could not observe a bias for Swedish for this experiment.

For Italian, we further validated the experiment by replacing the word *dirigente* with the word *successo*, which does not refer to a person. We could confirm the bias with a *p*-value of 0.01467 and an effect size of 3.133.

Using the keywords regarding math/arts and male/female words from the WEAT7 experiment, we could identify a bias for Italian and Swedish. For the experiment WEAT8, where science/arts and male/female words were considered, a bias was detected for Swedish, but not for Italian. In our previous work, both WEAT7 and WEAT8 were not confirmed for German and French word embeddings. Table 7 shows the results of the experiments, reporting the *p*-values and the absolute value of the effect size.

**Table 7:** Results of the validation for Italian and Swedish.

**Experiment**	***p*-value**	**Effect size d**	**Bias detected?**
WEAT5-ita	0.01209	0.870561014	✓
WEAT6-ita	0.01865	3.084297864	✓
WEAT6-ita-ch	0.00115	3.136910288	✓
WEAT7-ita	0.04995	0.155938722	✓
WEAT8-ita	0.4916	0.505244366	×
WEAT5-swe	0.0396	1.575493266	✓
WEAT6-swe	0.12559	3.74003113	×
WEAT7-swe	< 10^−3^	0.23436922	✓
WEAT8-swe	0.00185	0.460947275	✓

*We report p-values (p) and absolute value of effect size (d)*.

### 3.2. Origin Bias in Switzerland

In our new word sets investigating an origin bias toward particular groups of the population in Switzerland, a statistically significant bias was shown for all the three experiments. It was shown that there is a bias regarding pleasant/unpleasant words for particular groups of the population present in Switzerland, comparing typical Swiss names to names of different origin considering the most common non-EU origin countries of Swiss residents (*WEAT5-origin*). In particular, the experiments investigating on the bias concerning labor market and social integration could be confirmed for both women and men (*WEAT6-origin-m/f* ). Table 8 shows the results, reporting the *p*-values and the absolute value of effect size.

**Table 8:** Results of the new experiments investigating origin bias in Switzerland on German word embeddings.

**Experiment**	***p*-value**	**Effect size d**	**Bias detected?**
WEAT5-origin	0.00192	1.027370342	✓
WEAT6-origin-m	0.00026	0.021395449	✓
WEAT6-origin-f	0.00302	0.047869917	✓

*We report p-values (p) and absolute value of effect size (d)*.

### 3.3. BiasWords–Newly Detected Word Sets

Based on the application of BiasWords to the previously identified word sets containing bias (i.e., WEAT 5 to 8, and GER-1/GER-2; Kurpicz-Briki, 2020), we identified the word sets shown in Table 9 exposing a statistically significant bias.

**Table 9:** Results of the new word sets created with the BiasWords method and its validation using the WEAT method.

**Group**	**BIASW1**	**BIASW2**	**BIASW3**
Group 1	Mikrosensorik, Verkehrsingenieurwesen, Mikrosystemtechnik, Experimentalphysik, Kommunikationsinformatik, Chemieingenieurwesens, Biophysik, Wirtschaftsingenieurwesens	Sternenkunde, Nuklearchemie, US-Weltraumbehörde, Techologie, Kernphysik, Goenner, Planetenbeobachtung, Amateurastronomie	Handlungswissen, Alltagswissen, Menschengeist, Moralität, Lebenshauch, Instinkt, Seele, Hirn
Group 2	Pferdemedizin, Ernährungswissenschaften, Entwicklungswissenschaft, Bildungswissenschaft, Bildungswissenschaften, Tierärzte, Kleintiermedizin, Biopsychologie	Volkstanz, Drama, Nicht-Kunst, poetischen, Getanzte, Hip-Hop-Tanz, Comicliteratur, Liebesroman	Gläubigkeit, Affektion, Wahrnehmungsleistungen, Verständigkeit, Sensibilität, Abstraktionsfähigkeit, Volksreligiosität, Wahrnehmungskompetenz
Target 1	Onkel, Bauernjunge, Knabe, Jugendlicher, Jugendliche, Eheman, Schwiegervater, Stiefsohn	Onkel, Cousin, Enkelsohn, Kumpel, Opa, Urgrossonkel, Grossvater, Schulfreund	Jugendlicher, Sohn, Schwager, Schwiegervater, Neffe, Bruder, Schwestersohn, Bauernjunge
Target 2	Halbschwester, Teenager, Christine, Partnerin, Lebensgefährtin, Ärztin, Enkelin, Geschwister	Tochter, Enkeltochter, Ur-Grossmutter, Gattin, Mitschwester, Eltern, Nachbarin, Schwägerin	Freundin, Jungen, Baby-Mädchen, Tochter, Frau, Mutter, Kollegin, Mächen
*P*-Value	0.00757	0.04704	0.00192
Effect Size (d)	2.17228637	0.71880645	0.48409739

*We report p-values (p) and absolute value of effect size (d)*.

The word set *BIASW1* is based on the original word set *GER-1*, which contains common study choices in Switzerland with a majority of men and women respectively, and male and female words. The new words identified for the study choices include other related subjects for the male dominated word set, for example, different forms of engineering or physics (the original word set included *electrical engineering, mechanical engineering, computer science, microtechnology, and physics*). We can observe similar words in the female dominated study choices, whereas the original word set contained *veterinary medicine*. Here we, find additional bias for subjects such as *Pferdemedizin (engl.: horse veterinary)*.

The word set *BIASW2* is based on the original word set *WEAT 8*, which compares science and arts words to male and female words. More specific words have been identified for the domain arts (e.g., *Volkstanz*, engl.: *folklore dance*) and science (e.g., *Kernphysik*, engl.: *nuclear physics*).

The word set *BIASW3* is based on the original word set *GER-2*. The GER-2 word set is based on stereotypes of men and women of the 18th century, based on a historical document. Therefore, in this study, we observe words such as *Handlungswissen* and *Alltagswissen* referring to knowledge, and words such as *Sensibilität* and *Wahrnehmungskompetenz* referring to social skills and emotions.

## 4. Discussion

In this section we discuss potential reasons to explain the results and investigate implications of the results for the field of research.

### 4.1. Measuring Bias in Regional Languages

We investigated also on bias detection on regional languages—in particular for Romansh. However, the word embeddings for Romansh are based on a very small number of available articles (see Table 1). This is due to the small number of native speakers of this language. Even though it is one of Switzerland's national languages, only 0.5% of Swiss people listed Romansh as one of their main languages in 2013, and speakers are typically bilingual with at least another official language^14^. It is a very regional language and spoken mainly in the canton of Graubünden. Due to this, the available content to train word embeddings is rather limited and, since the language is mainly kept as a cultural heritage, we assume that the diversity of contents may also be restricted, as compared to the other languages. Therefore, only one of five experiments on the Romansh word embeddings identified a bias. We, therefore, argue that the topics of the training data and the available quantity of content for word embeddings of local languages leads to less bias within them.

### 4.2. Multi-Lingual Gender and Origin Bias

The origin bias investigated in the original WEAT5 experiment on English word embeddings, and previously confirmed for German and French, could also be confirmed for Italian and Swedish. The results indicate that this form of bias is universal and appears in different languages in a similar fashion, based on first names of minorities in the population.

The WEAT6 experiments considering family and career words and male/female names were confirmed for Italian but not for Swedish. Previously, we have shown that this bias exists in German word embeddings, and partially in French word embeddings (Kurpicz-Briki, 2020). One potential explication could be the view of women in business among these countries. For example, the percentage of mother's working full-time in Sweden is 83%, as compared to 55.7% in France, 30% in Germany, and 34.8% in Italy (Bahle, 2017). However, even though the bias concerning business and family tasks could not be confirmed, an other work has shown that gender-related stereotypes for specific professions do exist in Swedish word embeddings. For example, it has been shown that jobs such as civil engineer, statesman, or physics teacher are attributed to men, whereas midwife, housemaid, and nurse are assigned to women, for both GloVe and fastText word embeddings (Précenth, 2019). The study by Sahlgren and Olsson (2019) has also investigated gender-specific professions in Swedish word embeddings and came to the conclusion that the most common occupations for men/women that are statistically seen in Sweden are also reflected in fastText word embeddings. Interestingly, word2vec word embeddings did not show the same bias. The study by Biasion et al. (2020) on the Italian language has shown the presence of bias in job-related word embeddings.

The experiments concerning science/arts and math/arts words in combination with male/female words obtain different results for different languages. In particular, in our previous work for German and French (Kurpicz-Briki, 2020), these originally English experiments could not be confirmed. While the results from English could be confirmed for Swedish, where both WEAT7 and WEAT8 showed a bias, they were only partially confirmed for Italian. One potential explanation for this could be the type of language: Swedish, German, and English are Germanic languages, whereas French and Italian are Romance languages. The exception would, in this case, be German, but here the wording had to be modified due to specialities of the language (see Kurpicz-Briki, 2020 for details), which might have impacted the results.

In the proof of concept for the BiasWords method, we used a random composition of new words and still identified several new word sets showing additional forms of bias in word embeddings. This indicates that such approaches exploring the neighboring vector space of words containing bias is promising and should be further explored in future work.

### 4.3. Implications of Cultural Differences in Bias

Stereotypes, based on different factors such as profession, nationality, or origin, are highly problematic because they can change the behavior toward the concerned individuals (Spencer et al., 2016). It has been shown that such stereotypes are reflected in word embeddings (e.g., Bolukbasi et al., 2016; Caliskan et al., 2017). The bias in word embeddings goes even beyond this: research has proven that human stereotypes concerning personality traits can be identified in word embeddings (Agarwal et al., 2019). The authors collected human judgments about a person's Big Five personality traits (John et al., 1999) based on information on occupation, nationality, or description of the person. This documented human bias is then shown to be also present in lexical representations.

As a matter of fact, these human stereotypes may vary among different cultures, countries, or languages. For example, a study investigated how different language guidebooks on Switzerland differ in their way of representing the country (Bender et al., 2013). The authors found that there are differences on how aspects such as for example language skills, nightlife habits, or discipline of the Swiss population are described in different languages. It has been shown that some patterns of stereotypes are cross-cultural (sexism and ageism), whereas others stereotypes are more culture specific and variable (ethnicity, race, and religion), being based on the cultural context and related to history (Fiske, 2017).

Similarly, our work indicates that the stereotypes encoded in word embeddings depend on a linguistic and cultural context and must, therefore, be carefully considered for different languages. The way such stereotypes trigger a different behavior in a person, and we assume also in an automatic system relying on word embeddings, is for example a specific name. It has been shown with field studies that depending on origin, a discrimination on the labor market for specific groups can be identified (Koopmans et al., 2018). In particular, such studies have sent out the same job application with different names to current job offers and identified a difference in success between local names and names of foreign origin (Schneider et al., 2014). This discrimination based on the origin of a person's name can also be confirmed for word embeddings by the results of our work. Our results have shown that this is in particular true for German word embeddings and three groups of population in Switzerland of different origin. We identified a statistically significant bias between common Swiss names and common names originating from Kosovo, North Macedonia, and Turkey and positive and negative words. In particular, we also considered the situation of the labor market and showed a statistically significant bias regarding the same names considering words related to professional success and failure.

Such stereotypes have direct implication on the daily life of the concerned individual. When sending job applications, such implicit bias in humans leads to less invitations for interviews. But what is the impact of such stereotypes encoded in word embeddings on the natural language processing applications using them?

It has been proven that classifiers trained on biased word embeddings replicate the bias encoded in the word embeddings (Papakyriakopoulos et al., 2020). The authors trained a sentiment classifier on word embeddings that had before been proven to be biased. The results show that the predicted score for stereotypical names of different populations reflected the bias in the underlying word embeddings. Similar results were obtained for male and female names. It is, therefore, crucial to mitigate the bias and avoid further replication at the level of the application.

It also been shown that gender bias is a problem in search engine ranked lists. The Gender Stereotype Reinforcement (GSR) (Fabris et al., 2020) measure quantifies the tendency of such a ranked list to support gender stereotypes. To detect and quantify the extent of this, the GSR measure exploits gender bias encoded in word embeddings.

Different methods have been proposed to investigate the origin of bias in word embeddings and mitigation measures. This mitigation can happen at different levels, including the original training data before training the word embeddings, debiasing the word embeddings itself, or using methodologies to debias the classifiers or application using the biased word embeddings. For example, it has been shown that by identifying specific resources from the training data, and removing them before training the word embeddings the bias can be reduced (Brunet et al., 2019).

Several methods for debiasing word embeddings have been proposed, for example, post-processing methods (Bolukbasi et al., 2016) or integrated into the training procedure of word embeddings (Zhao et al., 2018) to reduce gender bias. However, it has been argued that such methods do rather cover the bias and not remove it completely from the word embeddings (Gonen and Goldberg, 2019). This example, only considering the mitigation of gender bias, illustrates the complexity of the problem. Different forms of bias that can be mitigated at different levels and concerning different types of word embeddings and other language models make a general resolution of the problem complex. Other findings indicate that debiasing methods using an explicit set of words are unlikely to be effective (Agarwal et al., 2019).

Other approaches consider a mitigation at the level of the application that is using the word embeddings. For example, it has been shown that the mitigation at the level of a classifier that was trained on biased word embeddings was efficient (Papakyriakopoulos et al., 2020). The authors claim that the classifier learns further associations between the vectors, which are not considered when debiasing at the level of the word embeddings.

Research has clearly proven different types of bias (nationality, origin, gender,and personality) in different types of word embeddings and language models. It has been confirmed in this work that bias can come in different forms for different cultural and linguistic contexts, as it was assumed by previous research. However, a general estimation of the impact on how this bias is replicated in applications, and general mitigation measures to avoid this, is still an unsolved problem. Based on our research, we suggest future work to investigate approaches addressing the specific types of bias and consider bias mitigation for specific languages or cultural contexts separately.

## Data Availability Statement

The original contributions presented in the study are included in the article/supplementary material, further inquiries can be directed to the corresponding author/s.

## Author Contributions

Both authors listed have made a substantial, direct and intellectual contribution to the work, and approved it for publication.

## Conflict of Interest

The authors declare that the research was conducted in the absence of any commercial or financial relationships that could be construed as a potential conflict of interest.

## Footnotes

^1^https://fasttext.cc/docs/en/crawl-vectors.html

^2^https://meta.wikimedia.org/wiki/List_of_Wikipedias

^3^https://commoncrawl.github.io/cc-crawl-statistics/

^4^https://www.istat.it/it/dati-analisi-e-prodotti/contenuti-interattivi/contanomi

^5^https://www.paginainizio.com/nomi/classifica_nomi.php?ord=click&t=s

^6^https://www.scb.se/hitta-statistik/statistik-efter-amne/befolkning/amnesovergripande-statistik/namnstatistik/pong/tabell-och-diagram/nyfodda–efter-namngivningsar-och-tilltalsnamn-topp-100/pojknamn/

^7^https://www.bfs.admin.ch/bfs/it/home/statistiche/popolazione/nascite-decessi/nomi-svizzera.html

^8^https://www.scb.se/hitta-statistik/statistik-efter-amne/befolkning/amnesovergripande-statistik/namnstatistik/pong/tabell-och-diagram/nyfodda–efter-namngivningsar-och-tilltalsnamn-topp-100/pojknamn/

^9^https://www.bfs.admin.ch/bfs/de/home/statistiken/bevoelkerung/migration-integration/auslaendische-bevoelkerung.html

^10^Kosovo: https://ask.rks-gov.net/media/3820/names-and-surnames.pdf

^11^North Macedonia: https://forebears.io/north-macedonia/forenames

^12^Turkey: https://web.archive.org/web/20140714133216/http://www.nvi.gov.tr/Files/File/Istatistikler/Isimler/en%20çok%20kullan1lan%20ad.pdf

^13^https://www.bfs.admin.ch

^14^https://houseofswitzerland.org/swissstories/society/allegra-look-romansh-switzerlands-fourth-language
